# Correction: Ciprofloxacin-loaded bioadhesive hydrogels for ocular applications

**DOI:** 10.1039/d5bm90097b

**Published:** 2026-02-02

**Authors:** Islam A. Khalil, Bahram Saleh, Dina M. Ibrahim, Clotilde Jumelle, Ann Yung, Reza Dana, Nasim Annabi

**Affiliations:** a Department of Chemical Engineering, Northeastern University Boston MA USA; b Brigham and Women's Hospital, Harvard Medical School Boston MA USA; c Harvard-MIT Division of Health Sciences and Technology, Massachusetts Institute of Technology Cambridge MA USA; d Department of Pharmaceutics, Misr University of Science and Technology 6th of October City 12566 Giza Egypt; e Energy Materials Laboratory, School of Sciences and Engineering, The American University in Cairo New Cairo 11835 Egypt; f Massachusetts Eye and Ear, Department of Ophthalmology, Harvard Medical School Boston MA USA; g Department of Chemical and Biomolecular Engineering, University of California, Los Angeles Los Angeles CA 90095 USA nannabi@ucla.edu +1(310) 267-5927

## Abstract

Correction for ‘Ciprofloxacin-loaded bioadhesive hydrogels for ocular applications’ by Islam A. Khalil *et al.*, *Biomater. Sci.*, 2020, **8**, 5196–5209.

The authors regret an error in Fig. 6 in the original manuscript. The correct version of Fig. 6g–l is as shown herein. The authors confirm that this correction does not affect the data interpretation or conclusions of the original study.

**Fig. 6:**
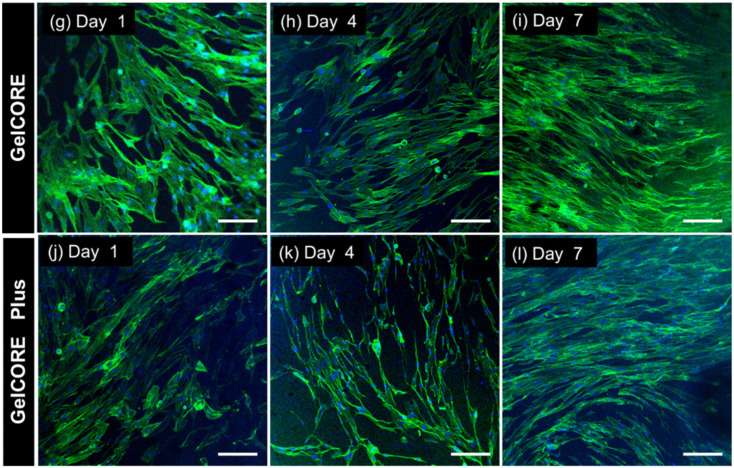
*In vitro* cytocompatibility of GelCORE + MCs bioadhesive. Representative Actin/DAPI images from corneal fibroblast cells on days 1, 4, and 7 after seeding (scale bar, 200 µm) on (g–i) GelCORE, and (j–l) GelCORE + MCs.

The Royal Society of Chemistry apologises for these errors and any consequent inconvenience to authors and readers.

